# Short‐Term Complications Are Rare After Cholesteatoma Surgery

**DOI:** 10.1111/coa.70111

**Published:** 2026-04-21

**Authors:** Agnes Modée Borgström, Cecilia Engmér Berglin, Johan Knutsson, Åsa Bonnard

**Affiliations:** ^1^ Department of Clinical Sciences, Intervention and Technology Karolinska Institute Stockholm Sweden; ^2^ Medical Unit of ENT, Hearing and Balance Karolinska University Hospital Stockholm Sweden; ^3^ Department of Otorhinolaryngology Sophiahemmet Hospital Stockholm Sweden; ^4^ Department of Otolaryngology Västerås Hospital Västerås Sweden; ^5^ Region Västmanland Uppsala University, Centre for Clinical Research, Västmanland Hospital Västerås Sweden

**Keywords:** cholesteatoma, infections, mortality, postoperative complications, thromboembolism

## Abstract

**Objective:**

To evaluate the incidence and nature of short‐term complications following cholesteatoma surgery in a heterogeneous cohort.

**Design:**

A retrospective cohort study including complete coverage of cholesteatoma surgery in a Swedish region between 1 January 2005 and 31 December 2015. A total of 966 patients were followed for 6 weeks postoperatively. Severe complications were defined as facial palsy, thromboembolic events, cerebrospinal fluid (CSF) leakage, serious postoperative infection, or death within 6 weeks of surgery.

**Results:**

Severe complications were uncommon, occurring in 14 patients (1.4%). Thromboembolic events were observed in five patients (0.5%), two of which (0.2%) resulted in postoperative mortality—one following cardiac arrest in the postoperative care unit and one due to a thromboembolic event approximately 3 weeks after surgery. Serious infections occurred in five patients (0.5%): three retroauricular abscesses, one case of meningitis, and one infected preauricular fistula with abscess formation. Additional severe complications included three cases of facial palsy and one case of CSF leakage.

**Conclusion:**

Cholesteatoma surgery demonstrates a low rate of severe short‐term complications, underscoring its overall safety with risks mainly attributable to general surgical or medical causes rather than cholesteatoma‐specific factors. The present cholesteatoma‐specific data constitute a solid basis for preoperative counselling and benchmarking within otology.

## Key Points

- Severe complications following cholesteatoma surgery occurred in 1.4% of patients.
- Postoperative mortality within 6 weeks after cholesteatoma surgery occurred in 0.2% of patients.
- A cohort with high completeness, representing a large spread of severity of cases, surgical methods and level of surgical education.

## 1 Introduction

Cholesteatoma surgery carries a risk of various complications, including hearing loss, taste disturbances, facial palsy, vertigo, and local or intracranial infections [1]. Previous studies on complications following cholesteatoma surgery have focused on comparing different surgical techniques [2, 3, 4, 5]. Some have studied specific types of cholesteatomas, for example, cholesteatomas with an intact ossicular chain, cerebellopontine angle cholesteatomas, or bilateral congenital cholesteatomas [2, 5, 6]. Others have studied long‐term results including the incidence of residual or recurrent cholesteatomas as well as hearing outcomes [7, 8, 9, 10]. The relevance of evaluating severe general surgical complications specifically in cholesteatoma surgery lies in addressing an evidence gap, as prior studies focus largely on broader surgical fields.

The incidence of severe general surgical and anaesthesiologic complications, such as death, cardiac events or thromboembolic events, varies in previous studies [11, 12]. However, patients undergoing otolaryngologic surgery do not appear to be at a higher risk of thromboembolic events or cardiac arrest compared to patients undergoing other surgical procedures [13, 14, 15]. Variability in surgical results, particularly when adverse events cluster, may have implications for both surgical development and patient safety. In this context, it is important to consider the incidence of severe general surgical and anaesthesiologic complications. To our knowledge, no previous studies have specifically examined the incidence of these complications following cholesteatoma surgery.

## 2 Objectives

The present study aimed to assess the occurrence of short‐term complications following cholesteatoma surgery in a heterogeneous cohort from a tertiary teaching hospital serving an entire region. Encompassing a broad patient population and surgeons with varying levels of experience, as well as different surgical techniques. This diversity enhances the generalizability of the findings and reflects real‐world clinical practice. The results are presented to aid clinicians in their preoperative decision‐making based on a large clinical dataset of consecutive patients.

## 3 Method and Materials

This was a retrospective cohort study including complete coverage of cholesteatoma surgery in a Swedish region between 1 January 2005 and 31 December 2015. Patients were identified by surgical and diagnostic codes for cholesteatoma, Appendix A. To avoid selection bias, only the first ear undergoing cholesteatoma surgery during the study period was included. If the patient had subsequent cholesteatoma surgery of the contralateral ear or had cholesteatoma surgery repeatedly on the ipsilateral ear, this surgery was not classified as a new case. Cholesteatoma classification was done according to the STAM‐system as described in the EAONO/JOS Joint Consensus Statement [16].

Follow‐up time was 6 weeks after surgery. Patients who had no follow‐up within 6 weeks were excluded. All medical records from our institution were carefully evaluated by the first author with a focus on pre‐, peri‐ and postoperative notes. If the information regarding the surgical procedure was unclear, an additional evaluation was performed by the last author, an experienced otologist. Severe complications were defined as death, thromboembolic events, serious infections, facial palsy or cerebrospinal fluid (CSF) leakage within 6 weeks following surgery. Serious infections were defined as intracranial infections, abscesses, and infections that were treated with intravenous antibiotics or surgical intervention.

Although a multivariate regression analysis was initially intended, the limited number of cases with severe postoperative complications necessitated the use of a univariate regression analysis. A 95% confidence interval was used, and statistical significance was set to *p* < 0.05. All statistical analyses were done using Microsoft Excel Version 16 and IBM SPSS Statistics Version 29.

Ethical permission was obtained by the Swedish Ethical Review Authority (2020‐05935), and informed consent was waived in accordance with applicable regulations. Reporting follows the Strengthening the Reporting of Observational Studies in Epidemiology (STROBE) guidelines. Microsoft Copilot has been used for language support.

## 4 Results

A total number of 981 patients underwent cholesteatoma surgery during the study period. Two patients were excluded due to missing data in the surgical records and 13 were lost to follow‐up. Thus, 966 patients were included in the study, and 237 patients (24.5%) were under the age of 18 at surgery. In total, 192 of the 966 patients had some kind of complication. Ten patients had two or more complications. Listed in Table 1 are pre‐ and perioperative characteristics of the patients included.

**TABLE 1:** Descriptive characteristics of the patients included in the study who underwent surgery for cholesteatoma between 2005 and 2015.

Total number of patients, *N*	966
Age at surgery, mean (SD)	35.3 (20.2)
Age range, years	2–95
Patients < 18 years, *N* (%)	237 (24.5)
	*N* (%)
Male sex	561 (58.1)
Left sided	501 (51.9)
Facial malformation^a^	40 (4.1)
Congenital cholesteatoma	22 (2.3)
	*N* (%)
Preoperative treatment for infection	504 (52.2)
Preoperative vertigo	52 (5.4)
Preoperative facial palsy	9 (0.9)
Previous ipsilateral ear surgery^b^	184 (19.0)
Previous ipsilateral cholesteatoma	125 (12.9)
Grommets	200 (20.7)
Ear infected at surgery	40 (4.1)
One middle ear site affected	205 (21.2)
Mastoid involvement	474 (49.1)
Three or more sites according to STAM affected	383 (39.6)

^a^ Facial malformations (*N*): Down syndrome (15), Orofacial cleft (15), Turner syndrome (2), Charge syndrome (1), Choanal atresia (1), Curry‐Hall syndrome (1), Fistula in the styloid process (1), Hemifacial microsomia and microtia (1), Marfans syndrome (1), Micrognathia (1), Möbius syndrome (1).

^b^ Myringoplasty, Ossiculoplasty, Cholesteatoma or Mastoidectomy.

In 461 patients a mastoidectomy was performed, in 78 patients the posterior wall was removed and in a total of 819 patients an endaural atticoantrotomy was described. However, in 132 patients the technique used was none of the here mentioned or unclear from the surgical charts. No endoscopic surgery of cholesteatoma was performed at the department during this time. A total of 22 surgeons of varying experience performed cholesteatoma surgery during the study period.

Severe complications occurred in 14 patients (1.4%), including two cases (0.2%) of postoperative mortality. One of these was due to a myocardial infarction with cardiac arrest in the postoperative care unit and the other due to a thromboembolic event about 3 weeks after surgery. Three patients had some kind of non‐lethal thromboembolic event postoperatively, Table 2. These included one case of pulmonary embolism, one case of deep vein thrombosis, and one case of ischemic stroke, and occurred between 3 days and 3 weeks postoperatively. The patients' ages ranged from 29 to 77 years. Both deceased patients and two of the patients that had non‐lethal thromboembolic events had some kind of risk factor: smoking, obesity, hypertension, previous stroke, and/or contraceptive treatment. One patient that had a non‐lethal thromboembolic event had received a single dose of thromboprophylaxis; the others did not receive any thromboprophylaxis. One of the deceased patients had paused their low‐dose Aspirin treatment, and it remains unclear whether it had been restarted postoperatively.

**TABLE 2:** Complications within 6 weeks of follow‐up.

	*N (%)*
Infections, total	121 (12.5)
Local infection	116 (12.0)
Serious infection	5 (0.5)
Vertigo	48 (5.0)
Bleeding or hematoma	6 (0.6)
Sensory desensitisation of extremities	5 (0.5)
Thromboembolic events, total	5 (0.5)
Mortality from thromboembolic event	2 (0.2)
Facial palsy	3 (0.3)
Cerebrospinal fluid leakage	1 (0.1)
Nosebleed	1 (0.1)
Severe headache	1 (0.1)
Thrombophlebitis	1 (0.1)
Tonsillitis	1 (0.1)
Urinary tract infection	1 (0.1)

Serious infections occurred in five patients (0.5%), including three cases of retroauricular abscesses, one case of meningitis, and one case of an infected preauricular fistula with the formation of an abscess. All these patients had a preoperative infection but only one was infected at surgery. Preoperative infection was defined as an infection treated with local, oral or intravenous antibiotics at any point preoperatively and mentioned in the medical charts. The less serious infections (*N* = 116 patients, 12%) include all cases described in the medical records as postoperative infections of the outer ear canal or retroauricular skin, and cases where local or oral antibiotics were administered, Table 2.

The univariate logistic regression analysis showed associations between postoperative complications of any kind and preoperative infection (OR 1.6, 95% CI 1.2–2.3), perioperative infection (OR 2.3, 95% CI 1.2–4.4), and mastoid involvement of the cholesteatoma (OR 1.9, 95% CI 1.4–2.6), Table 3. It also showed associations between serious postoperative infection and preoperative infection (OR 2.2, 95% CI 1.5–3.3) as well as mastoid involvement (OR 1.7, 95% CI 1.1–2.5), Table 3. It also showed that children < 12 years have less complications of any kind (OR 0.6, 95% CI 0.4–1.0), Table 3.

**TABLE 3:** Univariate logistic regression of the associations between postoperative complications within 6 weeks and pre‐/perioperative characteristics of cholesteatoma surgeries (*N* = 966) analysed with.

	Infection^a^ *(N* = 121)				Any complication (*N* = 192)				Two or more complications (*N* = 10)			
	*N* (%)	OR	95% CI	*p*	*N* (%)	OR	95% CI	*p*	*N* (%)	OR	95% CI	*p*
Age (< 12 years)	16 (13.2)	0.8	0.4–1.3	0.362	21 (10.9)	0.6	0.4–1.0	**0.033**	0 (−)	—	—	—
Sex (male)	72 (59.5)	1.1	0.7–1.6	0.718	113 (58.9)	1.0	0.8–1.4	0.807	4 (40.0)	0.5	0.1–1.7	0.255
Preoperative infection	83 (68.6)	2.2	1.5–3.3	**< 0.001**	119 (62.0)	1.6	1.2–2.3	**0.003**	6 (60.0)	1.4	0.4–4.9	0.620
Perioperative infection	6 (5.0)	1.2	0.5–3.0	0.634	14 (7.3)	2.3	1.2–4.4	**0.017**	0 (−)	—	—	—
Affection of the mastoid at surgery	73 (60.3)	1.7	1.1–2.5	**0.009**	119 (62.0)	1.9	1.4–2.6	**< 0.001**	7 (70.0)	2.4	0.6–9.4	0.202
Three or more STAM‐sites affected	51 (42.1)	1.1	0.8–1.7	0.554	87 (45.3)	1.3	1.0–1.8	0.074	7 (70.0)	3.6	0.9–14.0	0.065
Any previous ear surgery	42 (34.7)	0.9	0.6–1.3	0.430	68 (35.4)	0.9	0.6–1.2	0.430	2 (20.0)	0.4	0.1–1.9	0.257
Previous cholesteatoma surgery	16 (13.2)	1.0	0.6–1.8	0.932	31 (16.1)	1.4	0.9–2.2	0.142	1 (10.0)	0.7	0.1–5.9	0.745

*Note:* Bold indicates the statistically significant results.

^a^ Two patients deceased within 6 weeks of follow‐up were excluded in the analysis regarding postoperative infection.

Three patients (0.3%) had facial palsy that debuted postoperatively, Table 2. In two of these patients, the facial nerve was dehiscent at surgery. One patient with a dehiscent nerve at surgery had facial palsy directly postoperatively and the other had a delayed onset of facial palsy, with the debut 3 weeks after surgery. In the third patient, the facial canal was intact, and the symptoms debuted directly postoperatively. Three of the nine patients with preoperative facial palsy had full resolution of symptoms at 6 weeks of follow‐up.

One patient (0.1%) suffered from CSF leakage after surgery; this patient also had a severe headache but no signs of infection. Vertigo was mentioned in the medical records of 48 patients within 6 weeks of follow‐up, Table 2. Four out of these were diagnosed as benign paroxysmal positional vertigo; the rest were more diffuse in character.

## 5 Discussion

Cholesteatoma surgery demonstrates a low rate of severe short‐term complications, underscoring its overall safety with risks mainly attributable to general surgical or medical causes rather than cholesteatoma‐specific factors. These results can be used as reference material due to the high coverage and heterogeneity of the cohort.

In the present study, severe complications following cholesteatoma surgery were rare. The observed mortality, attributed to thromboembolic events, suggests a possible connection with the anaesthesia or duration of surgery rather than the surgical procedure. Reported postoperative mortality varies greatly in previous studies. Among healthy patients undergoing a wide variety of elective procedures it was 26.2 per million cases [11]. However, in patients > 70 years old undergoing non‐cardiac surgery with at least one night planned in hospital, there was a 5% mortality within 30 days of otolaryngologic and head neck surgery, in level with general surgery, gynaecological surgery and trauma surgery [12]. In the present study, mortality was 0.2%, which may be explained by the inclusion of all age groups, the non‐malignant nature of cholesteatoma, and the fact that cholesteatoma surgery generally constitutes a procedure with relatively limited tissue trauma. The present cholesteatoma‐specific data constitute a solid basis for preoperative counselling and benchmarking within otology.

Thromboembolic events are a recognized complication to surgery in general, with the majority occurring within 4 weeks postoperatively [17]. Previous findings indicate that postoperative thromboembolic events are not more common in ear surgery compared to other head and neck surgeries, both benign and malignant [14, 18]. Venous thromboembolism was shown to occur in 0.5% or less of patients undergoing ear surgery, in level with the present study [14, 18]. Thromboprophylaxis has been shown to have limited value in reducing thromboembolisms in otolaryngological patients but increasing the risk of bleeding [13]. Unfortunately, no data on thromboprophylaxis administration was available in the present study and no conclusion on their effect on thromboembolic events can therefore be drawn. At our department, massage of the legs was given routinely to patients undergoing cholesteatoma surgery; however, thromboprophylaxis was not administered routinely. Leg massage has been shown to reduce the risk of postoperative deep vein thrombosis [19]. This practice has not been changed following the results of the present study. At the department, the routine was, and still is, that patients return home either the same day or the day after cholesteatoma surgery; thereby, early mobilization is naturally promoted.

Postoperative infections were more frequent among patients with a history of preoperative infection, though not in those with an active infection at surgery. This could perhaps be explained by a more liberal administration of peri‐ or postoperative antibiotics when an active infection was present, compared to patients without a history of previous infection. In previous studies, patients undergoing cholesteatoma surgery have been shown to get postoperative infections, needing oral or intravenous antibiotics, in 4.5% of cases, and the rate was not affected by prophylactic postoperative antibiotics nor surgical method [20]. Postoperative infections both of the retroauricular skin and deeper surgical site infections occurred in 14% of patients undergoing mastoid and epitympanic obliteration to prevent retraction disease [4]. Postoperative infection within 6 weeks occurred in 8% of patients after myringoplasty for dry tympanic perforations, and no difference was seen between those who received prophylactic antibiotics and those who did not [21]. In the present study local infections occurred in 12% of patients and serious infections only in 0.5% of patients. The incidence of infections is difficult to compare due to varying definitions of infection [4, 20, 21]. Previous research has not demonstrated a benefit of prophylactic antibiotics which argue against routine administration of perioperative antibiotics [20, 21]. However, no data on antibiotic administration was available in the present study and therefore no conclusions can be drawn on this matter.

In the present study, data on operative time was unavailable and therefore no conclusion can be drawn on whether this affected the rate of infections in larger or more complex cholesteatomas. Regarding surgical complications, it is important to take into account that surgical treatment of cholesteatoma is unavoidable since cholesteatomas have a risk of serious complications [1].

Only 0.3% of patients had facial palsy that debuted postoperatively which is in the lower range of what can be expected, compared to a review where the incidence of postoperative facial palsy was 0.2%–1.9% [22]. There was also only one case (0.1%) of CSF leakage. The low rates of postoperative facial palsy and CSF leakage are positive but any conclusion on the association to cholesteatoma characteristics cannot be drawn due to the rarity of these events. A theory on why these complications were rare in the present study is that a large proportion of cholesteatomas in this cohort were treated at an early stage due to generally available and publicly funded health care.

## 6 Strengths and Limitations

No hearing assessment was done in the present study due to the short follow‐up. Hearing results have previously been evaluated at around 1 year after surgery [23]. Due to the short follow‐up, neither tinnitus nor taste loss was analysed.

The infrequency of severe complications in the present study limited our ability to perform comparative analyses across different subgroups of cholesteatoma patients. To the best of our knowledge there is no consensus on when to assess short term complications after cholesteatoma surgery. The 6‐week follow‐up period selected for the present study was intended to closely correlate the complications with the surgical procedure and anaesthesia. Additionally, the clinical routine includes a check‐up within approximately 6 weeks after surgery.

This was a retrospective cohort study investigating the occurrence of short‐term complications following cholesteatoma surgery in a heterogeneous cohort from a tertiary teaching hospital serving an entire region. Encompassing a broad patient population and surgeons with varying levels of experience, as well as different surgical techniques. Another strength of the study is that a detailed review of the pre‐, peri‐, and postoperative period was possible, and variables that are not present in the registers could be studied. However, there is a risk for faulty interpretation and registration of data from the medical records.

## 7 Conclusions

Cholesteatoma surgery demonstrates a low rate of severe short‐term complications, underscoring its overall safety with risks mainly attributable to general surgical or medical causes rather than cholesteatoma‐specific factors. The present cholesteatoma‐specific data constitute a solid basis for preoperative counselling and benchmarking within otology.

## Author Contributions

A.M.B., C.E.B., J.K. and Å.B. designed the work. A.M.B. acquired and analysed data with support from Å.B. A.M.B. drafted the manuscript. A.M.B., C.E.B., J.K. and Å.B. revised and approved the manuscript A.M.B., C.E.B., J.K. and Å.B. agree to be accountable for all aspects of the work.

## Funding

Grants from The Center for Innovative Medicine (FoUI‐975599), ALF (FoUI‐955027), the Swedish Hearing Research Foundation (HFF19‐0016) and ACTA Otolaryngologica.

## Disclosure

AI have been used for grammatical and spelling support.

## Ethics Statement

Ethical permission was obtained by the Swedish Ethical Review Authority (2020‐05935), and informed consent was waived in accordance with applicable regulations.

## Conflicts of Interest

The authors declare no conflicts of interest.

## Data Availability

The data are not publicly available due to privacy and ethical restrictions.

## Appendix A

In the present study cholesteatoma surgery was defined as having received one of the following surgical codes according to the Classification of Health Care Procedures: DCD20, DEA10, DEB00, DEB10, DEB20, DEB25 or DEB30 in combination with one of the ICD‐10 diagnostic codes: H71.9, H72.0, H73.8, H74.8 and H95.0.
